# Introduction to the special issue on analytical and decision-making technique innovation in financial market

**DOI:** 10.1186/s40854-020-00215-z

**Published:** 2020-11-19

**Authors:** Liang Xu, Liuren Wu, Xiao Li, Feng Shen

**Affiliations:** 1 Southwestern University of Finance and Economics, Chengdu, China; 2 City University of New York, New York, USA

The selection of appropriate analytical methods and techniques for decision-making is crucial in the financial markets, to correctly assess investments. Effective managers use a variety of decision-making techniques as they seek to evaluate alternatives and make sound decisions. After the COVID-19 pandemic, the global financial market has experienced sharp fluctuations. Recent technique innovations in the financial markets include both the development of new models as well as the incorporation of big data. In this special issue, we provide an overview of analytical and decision-making technique innovations arising from research and development. We also introduce the papers in this special issue. We show that global adoption of blockchain and big-data technologies would be the most successful technique in finance and other business sectors, and will lead to analytical and decision-making innovations as well as many research opportunities.

This special issue is the 24th issue of Financial Innovation (FIN), Volume 6, No. 6 (2020). It presents six papers contributed by 14 authors and co-authors from Brazil, China, Indonesia, Iran, Taiwan, and Turkey. The six papers explore contemporary theories or state-of-the-art technologies including, but not limited to, the following areas:

1. Predictive technique
  Nowadays, the predictive technique has been drawing attention in relation to emerging financial products such as bitcoin. The paper titled “Predicting changes in Bitcoin price using grey system theory” adopts the GM(1,1) model with a first-order differential equation to model the trend in time series.
2. Risk measure technique
  The performance of trading stocks immediately after sharp movements in exchange rates has seldom been explored in existing studies. In the paper titled “Trading stocks following sharp movements in the USDX, GBP/USD, and USD/CNY,” historical data of the constituent stocks of the DJ 30, FTSE 100, and SSE 50 indexes were analyzed, with the results indicating that the share prices were more volatile after sharp movements in the CNY, even though the CNY was less volatile given China’s exchange rate policy.
3. Effectiveness analysis technique
  Topics related to investment effectiveness, such as hedge effectiveness, have long been of interest in the financial literature. The main objective of the paper “Hedge effectiveness of put replication, gold, and oil in ASEAN-5 equities” is to analyze the effectiveness of put replication strategies, gold, and oil on hedge equities in the ASEAN-5 (Indonesia, Malaysia, Singapore, Thailand, and the Philippines). This study also implies that risk-averse investors should opt for a put-replication strategy or guaranteed financial products, rather than a commodities-hedging strategy. The paper titled “How to compare market efficiency? The Sharpe Ratio based on the ARMA-GARCH forecast” develops a formula of the Sharpe ratio from the ARMA-GARCH model and finds that the Sharpe ratio depends only on the coefficients of the AR and MA terms and is not affected by the GARCH process.
4. Pricing technique
  With the development of Bitcoin, Bitcoin pricing analysis has drawn increasing attention. The paper titled “Bitcoin pricing: Impact of attractiveness variables” seeks to contribute to Bitcoin pricing analysis based on the dynamics between variables of attractiveness and the value of the digital currency. This study demonstrates that, during a worldwide crisis, Bitcoin becomes an alternative investment, increasing its price. Based on this finding, Bitcoin may be used as a safe haven by the financial market and its intrinsic characteristics might help investors and governments find new mechanisms to deal with monetary transactions.
5. Knowledge-based decision-making
  Nowadays, an increasing number of knowledge-based decision-making methods are being employed in every aspect of the financial market for different scales such as micro (individual investment), medium (companies), and macro (central bank) decisions. The paper titled “Decision making on financial investment in Turkey using ARDL long-term coefficients and AHP” aims to solve the following decision-making problem: “Which financial investment instrument is most apt in the Turkish financial market?”. By using the coefficients obtained from the ARDL model and the analytic hierarchy process (AHP) model, the EURO was identified as the most appropriate investment instrument for individual investors.

